# Investigation of the Content Validity, Feasibility, Internal Consistency, and Construct Validity of 5 Patient-Reported Outcome Questions on Patient Involvement in Care Among Adolescents With Type 1 Diabetes: Multimethods Study

**DOI:** 10.2196/86580

**Published:** 2026-05-19

**Authors:** Annesofie Lunde Jensen, Rikke Bjerre Lassen, Caroline Bruun Abild, Kurt Kristensen, Lene Juel Solskov, Jens Thusgaard Hoerlück

**Affiliations:** 1 Clinical Medicine, Health, Aarhus University, Palle Juul Jensens Boulevard 11, Aarhus, 8200, Denmark, 45 20470260; 2 Steno Diabetes Center Aarhus, Aarhus University Hospital, Aarhus, Denmark; 3 Defactum, Aarhus University, Aarhus, Denmark

**Keywords:** children, patient involvement, indicator measures, patient-reported outcome, PRO, patient and public involvement, validation, cognitive interview, diabetes, adolescent

## Abstract

**Background:**

Validated measurement tools that focus solely on assessing adolescents’ involvement in their treatment are scarce.

**Objective:**

The study aimed to validate 5 patient-reported outcome (PRO) questions regarding patient involvement in care among adolescents with type 1 diabetes.

**Methods:**

A multimethod and questionnaire design was used. A total of 447 adolescents (aged 11‐18 y) completed a short questionnaire that included 5 PRO questions regarding patient involvement in their care. In total, 25 interviews were conducted with 20 adolescents, as some adolescents were interviewed twice. The content validity, feasibility, internal consistency, and construct validity of the PRO questions were analyzed.

**Results:**

Assessment of validity revealed that most participants encountered no or minor difficulties comprehending the PRO questions. The interviews demonstrated that all participants showed a satisfactory understanding of 3 of the 5 PRO questions. However, the terms “appropriate” and “experiences” posed a challenge for 3 out of 20 participants. Using data from the questionnaire, the feasibility, internal consistency, and construct validity analyses uncovered only 2 limitations in validity. First, the clinical use of the questions limits anonymity, potentially introducing bias. Second, adolescents younger than the age of 15 years had more difficulty with the item “I talked to the health care staff about the questions or concerns I had.”

**Conclusions:**

The PRO questions are valid for measuring patient involvement among adolescents with type 1 diabetes. Respondents stated that the PRO questions were easy to understand, relevant, and comprehensive. There should be awareness when using the PRO questions, as they are most robust for those aged 15 years or older. Therefore, using these PRO questions in both clinical settings and research demands thoughtful consideration regarding their application and setup, particularly when considering the age of respondents and circumstances. It is worth noting that these PRO questions may pose validity challenges for use among adolescents younger than the age of 15 years with type 1 diabetes.

## Introduction

Involving patients in their health care, for example, in planning and decision-making regarding their disease, has been shown to positively impact treatment outcomes [1,2]. This is also important for children and adolescents. Studies have shown that most adolescents want to take part in their care, but they can also feel pressured when involved in it [3]. Patient involvement refers to the patient’s rights and benefits from having a central position in their health care. Patient involvement builds on a patient-clinician partnership characterized by dialogue, mutual trust, respect, and shared decision-making [4]. According to the “Convention on the Rights of the Child,” adolescents have a right to express their opinions [5]. Given this, over the past 20 years, there has been an increased focus on the importance of effective communication and adolescents’ involvement in their health care [6,7]. When involving children and adolescents in their health care, it is important to consider the verbal and sociocognitive differences across this age group, as well as the sociocultural and clinical context. Studies have shown that these factors affect their ability to participate and self-report, especially before the age of 12 years but also between 12 and 18 years [8]. Studies show that adolescents with chronic diseases like diabetes want to be involved in their care [9]. Adolescents especially desire to gain more control over their lives by being more involved in decision-making about managing their illness [3,9]. Hence, it is important to involve adolescents with diabetes in their care, as their experiences and attitudes may affect the quality of care [10,11]. Despite this, including a patient-reported outcome (PRO) questionnaire of patient involvement in the development and evaluation of health care interventions, as well as in clinical practice, is still limited among adolescent health care [12]. Furthermore, PRO questions are equal to PRO measures, which are defined as any report of the status of a person’s health condition, for example, their general well-being or diabetes distress, that comes directly from the person without interpretation of their response [13]. This means that PROs are used as an umbrella term, involving both person-reported outcome measures, such as physical functioning and mental health, and person-reported experience measures, for example, communication with clinicians and partnership [13,14]. Based on the above, we define PRO questions of patient involvement as questions that reflect the degree to which the person feels involved in their care. Most questionnaires that incorporate adolescents’ viewpoints regarding their involvement in their care do so implicitly by asking about their condition and perceptions of the quality of care [12]. Concerning adolescents with diabetes, both generic and disease-specific questionnaires exist [15,16], which implicitly include this. This perspective is included in questionnaires, such as the Child Depression Inventory–Short [17], Barriers to Diabetes Adherence [18], Readiness for Emerging Adults with Diabetes in Youth [15], and World Health Organization–5 (WHO-5) Well-Being Index [19]. Other PRO questionnaires have incorporated one or more questions regarding involvement, along with questions focusing on symptoms or treatment outcomes [20]. A scoping review identified 23 PRO questionnaires used to measure different kinds of treatment involvement among adolescents with type 1 diabetes, for example, communication with clinicians, clinicians’ commitment, and shared decision-making. This included, for example, the generic questionnaire Chronic Illness, Resource Survey or the diabetes-specific Diabetes Family Conflict Scale [20]. Still, to our knowledge, no validated PRO questions precisely assess patient involvement among adolescents. Such PRO questions could be important to offer feedback to the clinical team and support the implementation of new involvement initiatives to meet the goals, as well as to uncover the effects of new patient-involving initiatives for adolescents. In an adult (aged >18 y) context, 5 PRO questions for patient involvement have been developed and validated [21]. These PRO questions can be used across patient groups and for various health services [21]. The PRO questions were developed to measure the degree of user involvement intervention and provide feedback to health care professionals regarding the quality of care [22]. Hence, they were developed as PRO questions to be used anonymously in research or development projects, not for clinical practice [23]. The development shows that the questions had a skewed distribution and high interitem correlation. Furthermore, the development and validation of the PRO questions showed that classic background variables such as gender, age, and education have a limited impact on patients’ responses. The most significant effect relates to the patients’ “self-perceived health”; the more ill a patient perceives themselves to be, the more critical they are regarding the experience of involvement. Furthermore, validating the significance of the background factors for how vital the 5 PRO questions are for different patient groups showed that age had a limited influence. In particular, the questions are less significant for patients older than 80 years. Additionally, there is a tendency for involvement to be more important for women than for men, and the longer the patient’s education, the more emphasis is placed on involvement. Also, there was no difference in the importance of the questions for patients with somatic and psychiatric conditions. Furthermore, it holds that the more involvement a patient desires, the more significant the involvement questions become. Taking this into account, we rely on the development and validation of the PRO questions among adults [21,22]. The content and wording of these PRO questions were deemed appropriate for assessing patient involvement in adolescents with diabetes (aged 11‐18 years) in a clinical setting. Although not originally developed for adolescents and not previously used as PRO questions in clinical practice [21], this study aims to validate the PRO questions in adolescents with diabetes. Furthermore, we hypothesize that validity may be affected by the adolescents’ differences in metabolic regulation, hemoglobin A1c (HbA_1c_), age, and gender, as well as their desire for involvement.

## Methods

### Design

This study uses a multimethod design, drawing on a questionnaire and interviews to collect data from adolescents with type 1 diabetes and secondary diabetes in an outpatient hospital setting. This design allows researchers to gather data at a single point in time, which is suitable for evaluating the psychometric properties of the questions, including internal consistency and construct validity. The study used the COSMIN (Consensus-Based Standards for the Selection of Health Measurement Instruments) [24] checklist (Checklist 1) and the analytical model from Murphy et al [25] study on the Primary Care Outcomes Questionnaire to select measurement instruments and guide reporting on measurement properties. Together, this is characterized by a psychometric approach designed to validate reliability, validity, and sensitivity in measuring user involvement. To establish content validity and identify and correct challenges, we conducted semistructured interviews [26] with adolescents with diabetes. The interviews were used to evaluate the quality of responses and to determine whether the questions elicited the information the author intended. This was combined with a statistical analysis of the 5 PRO questions. These analyses assessed feasibility using the amount and patterns of missing data and construct validity by testing prespecified hypotheses about the correlations between the questions. Through the analyses, we focused on age and gender, the potential effect of age on the pattern and content of participants’ responses, and, finally, whether the validity of the questions depended on age. Consequently, we performed all statistical analyses on the group, stratified by age (≥11‐15 y and >15‐18 y) and gender. In other words, the strength of the interviews is that they provide in-depth information regarding face validity in a narrow sample. In contrast, the strength of the statistical analyses lies in their ability to examine the broader population systematically. During the analytical process, we also involved patient and public (PP) partners.

### Ethical Considerations

The interview participants were informed verbally and in writing about the study. Written informed consent was obtained before the interviews. Permission to collect questionnaire data from the medical records was given by the Central Denmark Regional Legal Office (record 1-45-70-21-21). The study was registered in the Central Denmark Regional database (record 663160). All procedures performed were in accordance with the ethical standards of the National Research Committee and the Declaration of Helsinki [27]. The Science and Legal Office, Central Denmark Regional Office, assessed the study 1-16-02-326-19.

### Study Participants

The study was conducted among adolescents with type 1 diabetes and secondary diabetes aged 11 to 18 years, with a diabetes duration of more than 1 year. Secondary diabetes refers to diabetes that develops due to another medical condition or as a side effect of certain medications, rather than from autoimmune processes (as in type 1 diabetes). Examples of common causes of secondary diabetes include cystic fibrosis or medical treatment following a transplant. The participants came from 4 pediatric departments in the Central Denmark Region and were recruited between May 2019 and March 2022. All participants had internet access to answer the short questionnaire and could understand, read, and write Danish. For the first interviews, participants were purposively sampled to ensure variation in age and gender. The purposive sampling by gender was chosen because girls’ and boys’ physical, mental, and social development occur at different stages [28]. Convenience sampling was used for the second interview, and 5 prior participants were interviewed again. Participants for the interview came from the outpatient clinic at Steno Diabetes Center Aarhus, and the health care professional assisted in the recruitment.

### PRO Questions for Patient Involvement and Clinical PRO-Context

Before their annual consultation at the clinic, participants completed a short questionnaire assessing symptoms of disordered eating using the Diabetes Eating Problem Survey–Revised (16 items) [29,30], the generic WHO-5 Well-Being Index [19], patients’ overall satisfaction [31], and PRO questions for patient involvement [21]. The short questionnaire was part of standard care and was conducted using AmbuFlex telePRO solutions (Center for Patient-Reported Outcomes), a web-based platform for patients to answer PRO questions. When patients responded to the short questionnaire, their responses were directly accessible in their electronic health record [32]. The PRO questions consist of a 5-item questionnaire measuring the degree of agreement with statements on a 5-point scale, ranging from 0 (“Not at all”) to 5 (“A very high degree”), with an additional option to select “Don’t know” (Textbox 1). Multimedia Appendix 1 outlines the 5 PRO questions and the question concerning patients’ overall satisfaction, which was included in the validation. The question regarding patients’ overall satisfaction is also used in the Danish National Health Survey [31].

Textbox 1. **Indicator measurement regarding patients' experience of involvement (PRO: patient-reported outcome).**

These questions are about to what degree you have felt involved in your treatment? **(Select only one answer for each statement) [Disse spørgsmål handler om, hvordan du har følt dig medinddraget i din behandling? (sæt kun en markering ud fra hvert udsagn)]**

1. The healthcare staff inquired about my own experiences with my illness/condition. [Sundhedspersonalet spurgte ind til mine egne erfaringer med min sygdom/tilstand]
2. I talked with the healthcare staff about the questions or concerns I had [Jeg fik talt med sundhedspersonalet om de spørgsmål eller bekymringer, jeg havde]
3. The healthcare staff encouraged me to ask questions or talk about my concerns [Sundhedspersonalet opfordrede mig til at stille spørgsmål eller tale om bekymringer]
4. I was involved in the decisions about what was going to happen. [Jeg var med på råd, når der blev truffet beslutninger om det, der skulle ske]
5. I have had an appropriate number of conversations with the healthcare staff about how I can best handle my illness/condition. [Jeg har i passende omfang haft samtaler med sundhedspersonalet om, hvordan jeg bedst håndterer min sygdom/tilstand]
6. All in all, I am satisfied with my last visit to the outpatient clinic [Jeg er alt i alt tilfreds med mit sidste besøg i ambulatoriet]

Response categories: Not applicable, Not at all, To a slight degree, To some degree, To a high degree

To a very high degree, Don’t know [ Ikke relevant, slet ikke, I mindre grad, I nogen grad, I høj grad, I meget høj grad, Ved ikke]

The English translation of the Danish PRO questions [Danish version.], has been guided by the protocol and guidelines from the International Society for Pharmacoeconomics and Outcome Research (ISPOR) [33].

### Data Collection

Data collection was conducted iteratively and consisted of semistructured interviews and data from the short questionnaire described in the previous subsection.

The semistructured interviews were conducted from December 2020 to October 2021 (20 interviews) and from May 2022 to August 2022 (5 interviews). All 25 interviews were performed by the second author (20 interviews) or the third author (CBA; 5 interviews). The interviews took place in the clinic (n=2), by telephone at home (n=21), or via video (n=2). The interviews were audio-recorded and lasted between 11 and 48 minutes (mean 31 minutes). They were transcribed verbatim. A total of two semistructured interview guides were developed, focusing on (1) how participants constructed their answers, (2) what they interpreted the questions to mean, (3) whether they experienced any difficulties in answering, and (4) other aspects that shed light on the broader circumstances upon which their answers were based [26]. The first interview guide included four topics: (1) practical aspects of completing the short questionnaire (eg, “Tell me about the last time you completed the short questionnaire”), (2) short questionnaire content (eg, “How do you understand this question?”; selected questions from the short questionnaire were read to participants), (3) experiences with assessing their own condition and needs (eg,“What do you think about asking questions regarding the health care professionals’ ability to involve you in your treatment?”), and (4) use of the short questionnaire in consultations (eg, “Tell me about how the short questionnaire was used in the conversation with your doctor/nurse”) (Multimedia Appendix 1). The second interview guide focused solely on the use and value of the 5 PRO questions. These interviews were conducted to gather more data on participants’ experiences with the PRO questions. Participants were asked to express their understanding of all 5 PRO questions and explain what they meant in clinical practice. For example, they were asked what it would mean to “Have had an appropriate number of talks with the health care staff about how I can best handle my illness/condition.” Furthermore, participants were asked to describe whether they found it relevant to ask and use questions regarding adolescent user involvement (Multimedia Appendix 1). During the interviews, think-aloud and cognitive interview techniques were used. These are recommended techniques for investigating content validity [33,34]. The quantitative data for statistical analyses were collected from electronic health records in March 2022, covering participants’ data from May 2019 to February 2021. The data consisted of the 5 PRO questions and 1 question on the patient’s overall satisfaction, which was part of the short questionnaire used in participants’ standard care (described in the “PRO Questions for Patient Involvement and Clinical PRO-Context” section). Furthermore, the data included participants’ age, gender, HbA_1c_, treatment, duration of diabetes, and diabetes type.

### Data Analysis

The analysis of the PRO questions included content validity, feasibility, internal consistency, and construct validity.

Content validity was assessed through an analysis of all the interviews. This was an inductive process using the interpretive description methodology [35]. An important feature of this part of the analysis was the participation of PP partners in the process. The value of including PP partners in research is recognized and recommended, with benefits such as patient perspectives improving both research processes and outcomes to ensure they are patient-centered and focused on relevant patients’ needs [36,37]. The PP partners involved in the analytical process were 2 adolescents (one younger than 15 years and one older than 15 years) with type 1 diabetes. They were recruited from the adolescents’ user panel at the outpatient clinic at Aarhus University Hospital. They had experience with consultations at the outpatient clinic but no experience answering the questionnaires. Multimedia Appendix 2 outlines the PP partners’ participation in the analytical process.

The first part of the content validity analysis consisted of sorting data into broadly coded categories. The categories formed codes related to the 5 PRO questions, participants’ experiences with answering the short and PRO questions, how they valued the PRO questions, and challenges with using them. Further analysis investigated how the different statements were interlinked and highlighted similarities and differences within and between the interviews, looking for patterns, associations, and connections across the data. The first and last authors carried out this initial step. This process led to the first interpretation of the PRO questions’ validity. In this analysis stage, the PP partners were involved (Multimedia Appendix 2). The involvement of PP partners revealed no differences in the interpretation of the data. However, PP partners stressed that even though the participants understood the PRO questions, they preferred to use terms more commonly used by adolescents, for example, replacing “outpatient clinic” with the clinic’s name. After the involvement of PP partners, the results were discussed with the rest of the research team, and final interpretations were made, leading to a description of the dominant characteristics of how participants experienced the use and content of the PRO questions. NVivo (Lumivero) supported the organization, coding, and analytical queries across participants’ characteristics.

The COSMIN checklist guides the statistical analysis of the qualitative data, but some steps were omitted due to data limitations. The main limitation was that there was only 1 question per theoretical item. The statistical analysis focused on how age groups differentiated and was divided into those aged more or less than 15 years. The statistical analysis of structural validity tested 2 hypotheses using the Kendall τ-b correlation coefficient. The difference between the age groups is tested in the proportion missing by a 2-sample test for equality of proportions with continuity correction. The difference between means was tested with the Welch 2-sample 2-tailed *t* test. All analyses were performed using R version 4.0.2 (R Foundation for Statistical Computing).

## Results

### Participants

Questionnaires from 447 adolescents were included, and 20 of the adolescents participated in the interviews. Participants with secondary diabetes (n=3) were included due to their similar treatment approaches to participants with type 1 diabetes. Participants’ demographic and clinical characteristics are described in Table 1. Compared to the overall demographic population in the Central Denmark Region regarding age, gender, duration of diabetes, diabetes type, and HbA_1c_, there are no significant differences [38]. All participants in the interviews reported experience answering questionnaires in school or in a health care setting. They indicated it took 5 to 20 minutes to answer the short questionnaire. Half of the participants had responded to the questionnaires alone, while the rest had support from either parents or a health care professional.

**Table 1.** Demographic and clinical characteristics of the participants (N=447).

	Participants attending interview (n=20)	Participants answering questionnaires (N=447)
Age (y), mean (range)		
11‐18	15 (11-18)	14 (11-18)
Age (y), n (%)		
>11‐15	11 (55)	229 (51)
>15‐18	9 (45)	218 (49)
Gender, n (%)		
Woman	11 (55)	229 (51)
Man	9 (45)	218 (49)
Diabetes type, n (%)		
Type 1	18 (90)	444 (99)
Secondary diabetes	2 (10)	3 (1)
Duration of diabetes (y), n (%)		
<5	8 (40)	247 (55)
≥5	12 (60)	200 (44)
Diabetes treatment, n (%)		
Insulin pump	20 (100)	345 (77)
Sensor	20 (100)	102 (23)
HbA_1c_^a^ measurement, mean (range)		
HbA_1c_ (%)	7.0 (5.0-8.6)	7.6 (4.8-18.2)
HbA_1c_ (mmol/mol)	53.4 (31-71)	59.4 (29-175)
Context of treating hospital, n (%)		
Aarhus University hospital	20 (100)	216 (48)
Regional Hospital West Jutland	0 (0)	76 (17)
Regional Hospital Randers	0 (0)	62 (14)
Regional Hospital Viborg	0 (0)	93 (21)

a HbA_1c_: hemoglobin A1c.

### Questionnaire Responses

Of the 447 questionnaire responses, 258 (58%) respondents completed all involvement questions, while 189 (42%) were missing at least one of the involvement items. Responses such as “Don’t know” and “Not relevant” were treated as missing in the analyses. The indicators are individually scored from 1 to 5, with higher scores indicating greater involvement. As a total score, they are summarized into a simple summative index and rescaled to range from 0 to 100.

The preliminary analysis shows (Table 2) that each item has a mean above the middle of the scale and an SD of around 1 point. Together, these findings indicate that the scale of the answers is skewed, with responses concentrated at the positive end. It also shows that individuals aged older than 15 years have a significantly higher mean score on 3 of the 5 involvement items and the total index. When grouping by other factors, we observe no significant differences in gender, HbA_1c_ regulation, or WHO-5 Well-Being Index.

**Table 2.** General statistics about the items divided into age groups at age older than 15 years.

Item	Age group (y)						Difference	
	≥11‐15^a^			>15‐18^b^			*P* value missing	*P* value mean
	Missing (%)	Mean (SD)		Missing (%)	Mean (SD)			
The health care staff asked questions about my own experiences with my illness/condition	21.21	3.92 (1.01)		21.76	3.94 (0.96)		.89	.87
I talked to the health care staff about the questions or concerns I had	28.14	3.85 (1.19)		14.35	4.02 (1.07)		<.001	.16
The health care staff encouraged me to ask questions or talk about my concerns	18.61	3.54 (1.24)		15.74	3.99 (0.98)		.42	<.001
I was involved when decisions were made about what was to take place	13.42	3.76 (1.18)		12.50	4.13 (0.88)		.77	<.001
I have had an appropriate number of talks with the health care staff about how I can best handle my illness/condition	12.99	3.88 (1.07)		10.65	4.23 (0.78)		.44	<.001
Total involvement score (summative index of all 5 questions scaled 0‐100).	50	72.93 (20.81)		34	79.33 (16.14)		.12	.007

a Mean interitem correlation=0.490; Cronbach α=0.827.

b Mean interitem correlation=0.471; Cronbach α=0.815.

### Content Validity

Content validity is assessed through the analysis of the interviews, examining whether respondents understood the questions as intended. Most participants had no or only minor challenges in understanding the PRO questions. The PRO questions “The health care staff encouraged me to ask questions or talk about my concerns” and “I was involved when decisions were made about what was to take place” were understood by all participants. As one participant expressed regarding the first of the questions mentioned above, “Well, it’s like that if they ask you to ask questions or something like that” (ID 11).

In the PRO question, “I have had an appropriate number of talks with the health care staff about how I can best handle my illness/condition,” understanding the term “appropriate” was challenging for 2 participants (ID 1 and ID 2). Additionally, understanding the word “experiences” was challenging for 2 participants (ID 3 and ID 4) in the PRO question, “The health care staff asked questions about my own experiences with my illness/condition.” The PRO question, “I talked to the health care staff about the questions or concerns I had,” was understood by all the participants. However, many of them found it irrelevant to answer, as they described having no questions or concerns. Consequently, they answered, “Not relevant.”

Other words like “concerns” and “health care staff” were not a problem for the participants to understand, as 1 participant explained:

*I think using health care staff is fine because you do not specifically refer to a doctor. It may also be a nurse.*

[ID 5]

All participants appreciated being asked about their involvement in their care. As 1 participant explained:

*I think it’s pretty good because it just means that you care and that we feel good about coming to the hospital. It just shows that you're not indifferent, and you actually want to do something about it if there is something wrong.*

[ID 6]

Furthermore, being able to complete the questions before their consultation made them reflect more on their care and condition. They expressed that “you must be honest with yourself when answering the questionnaire because if you report a problem, you will be confronted with it during the consultation.” This was viewed positively, but also as a challenge that they believed health care professionals should be aware of:

*It can also be cool enough to have the opportunity to be honest. But I could also imagine that many would not answer them genuinely because it is very direct.*

[ID 5]

Since the questionnaire was completed before the consultation, participants had to recall and base their responses on the previous consultation 3 months earlier. Most participants expressed that this was a challenge. When asked by the interviewer, “When you answer the questions, what do you think back on?" 1 participant answered:

*“It depends on whether something specific happened. If a new choice was made about a new pump or… Then I might think back to the last check-up, but if it was just a check-up like the one or two times ago, then I think it’s just more general than I'm thinking.”*

[ID 10]

As a result, they described that their answers were often based on a more general impression of their clinic consultations.

### Feasibility

In the COSMIN framework, feasibility is defined as the ease of application and the availability of the measurement instrument. It is not considered a measurement property and is therefore not assessed using statistical methods; instead, it depends on the specific context of use. In this study, the response rate is used as an indicator of feasibility. Statistical analysis of 447 questionnaires from the short questionnaire showed that for participants aged between ≥11 to 15 years, the proportion of missing responses on the PRO “I talked to the health care staff about the questions or concerns I had” was over 30%, almost 50% higher than for participants aged between >15 and 18 years. This may be interpreted as a sign of difficulty answering the question, especially among the youngest participants.

### Internal Consistency

Internal consistency is defined as the degree of interrelatedness and is assessed using Cronbach α and the interitem correlation, and the mean interitem correlations were calculated as the average of the Pearson correlation coefficients between all item pairs. In both groups, α was well above the recommended threshold of 0.70, with no substantial differences observed between them (Table 2). For both groups, the mean interitem correlation was just below 0.5, which indicates adequate homogeneity among the items without suggesting substantial redundancy.

### Construct Validity

Construct validity refers to the degree to which scores align with theoretically derived hypotheses, including expected internal relationships, correlations with other instruments, and differences between relevant groups, under the assumption that the instrument validly measures the intended construct. Construct validity was assessed by testing a set of predefined hypotheses. These hypotheses were based on established properties of patient involvement, namely that higher levels of perceived patient involvement are positively associated with greater overall satisfaction and better self-rated health [39]. As expected, the actual Kendall τ-b correlations were strong for overall satisfaction in both age groups. For the WHO-5 Well-Being Index, the picture was different; the correlation was small for the youngest age group and insignificant. For the older group (aged >15‐18 y), the correlation was only in the expected direction but small with significance at the .05 level (Table 3).

**Table 3.** Expected and actual Kendall τ-b correlations with patient involvement.

Correlation	Age group (y)					
	≥11‐15		>15‐18		All	
	Overall satisfaction	WHO-5^a^ Well-Being	Overall satisfaction	WHO-5 Well-Being	Overall satisfaction	WHO-5 Well-Being
Expected correlations	+++^b^	+++	+++	+++	+++	+++
Actual correlations	0.457	0.067	0.52	0.122	0.425	0.091
*P* value actual correlation	<.001	.32	<.001	.05	<.001	.04

a WHO: World Health Organization–5.

b Strong correlation.

## Discussion

The PRO questions were originally designed to measure the degree of patient involvement in adult clinical interventions and to provide feedback to health care professionals on the quality of care. To our knowledge, this is the first study to validate PRO questions about patient involvement in adolescents with diabetes. Overall, the PRO questions proved valid for measuring patient involvement in adolescents with diabetes, as participants indicated that the PRO questions were easy to understand, relevant, and comprehensive. Some questions appeared harder for participants younger than 15 years to interpret, as indicated by a higher number of missing responses among these participants. If they did reply, there were no indications that their answers were less valid. Therefore, we conclude that although developed for assessing patient involvement in adults, the 5 PRO questions are also suitable, especially for adolescents older than 15 years. Younger adolescents had a larger number of missing responses, which could be due to problems with interpretation as well as developments in social and self-awareness [8]. However, certain adjustments and specifications regarding the target group, usage, and preparation could be considered to enhance the ability to measure adolescent patient involvement, emphasizing age and context.

We found that some participants had problems understanding specific item words, particularly the words “appropriate” and “experiences.” Other studies exploring the understanding of words among children and adolescents aged between 6 and 17 years have shown that these difficulties are related to age. It is incredibly challenging for those aged younger than 12 years [40,41]. Still, these studies also emphasize that some items may be too vague or ambiguous to be clearly understood. This may be the case with the words “appropriate” and “experiences.” Reviewing the items to discuss related synonyms and retesting them would be relevant. Another alternative would be to include instructions for the PRO questions before use. In our study, some participants relied on their parents to explain the words, leading to a more comprehensive understanding.

We found that the participants did not use the whole scale. The answers were concentrated on the positive end of the scale. Optimally, one would want the answers to be distributed symmetrically around the middle of the scale without a skew in any direction. On the surface, this suggests that patients have positive experiences with their involvement, but it may also reflect social desirability bias among patients. The biggest problem is that the distribution of responses is skewed toward the positive end of the scale. The skewed distribution reduces the question’s ability to discriminate and increases the risk of ceiling effects. This effect may limit responsiveness and the ability to detect improvements in longitudinal applications. Skewed distributions are a common problem in PRO questions, also known as person-reported experience measures [42].

This study measured the degree of involvement ahead of a consultation and as part of clinical practice. The participants described it as difficult to remember and evaluate the last consultation. Additionally, it could be challenging to honestly evaluate the health care professionals they were about to talk to. Still, the participants and the PP partners found it important to be asked these questions, as they stressed the importance of being involved—for example, being talked to rather than having the health care professional primarily address their parents. Other studies on diabetes care for adolescents describe similar results [43]. Hence, it is essential to have tools like these questions to measure patient involvement, support dialogue, and assess user involvement in clinical practice. The PP partners suggested that patient involvement questions could be handed out after the consultation, allowing adolescents to evaluate the consultation alone or with their parents. Testing different evaluations of user involvement in a clinical setting would be relevant to investigate further.

The PRO questions evaluated 5 dimensions covering adolescents’ involvement in care [4,21]. A scoping review of PRO questionnaires used to measure the experience of adolescents aged 11 to 18 years with type 1 diabetes and their involvement in treatment shows that it was common for other PRO questionnaires to include several dimensions of user involvement [44]. Still, none of the questionnaires focused solely on measuring user involvement while including other aspects of diabetes care. Access to validated instruments that focus exclusively on user involvement and encompass all aspects of user involvement, for example, exchanging information or involvement in decision-making, may contribute to a more stringent and focused evaluation of user involvement. Furthermore, as in other studies using the 5 PRO questions to investigate patients’ experience of user involvement, patients seem to report a relatively high score between “To a high degree” and “To a very high degree” [45,46]. One may argue that to represent a good score, the patient’s response should be either “To a high degree” or “To a very high degree.*”*

Using the PRO questions within a clinical environment, rather than their intended use in research or quality improvement, could potentially influence participants’ responses and introduce a risk of social desirability bias. This bias occurs when patients answer sensitive questions and choose what they believe is more socially desirable. In research and quality improvement, questionnaires can be completed after the consultation, and respondents are always anonymous. Hence, the participants in our study might have expressed a high degree of involvement because they knew that their health care professional would view their answers. The risk of this bias logically depends on 2 factors: first, whether the respondents consider the answer they want to give to be socially undesirable, and second, how much trust they have in their health care professional. This means we cannot rule out the possibility that the results of this study are context-dependent. In a different context, if questions were asked after the consultation or if there was lower trust between patient and clinician, the results might differ.

Structural analysis is understood as the degree to which the scores of a health-related PRO instrument adequately reflect the dimensionality of the construct being measured. It is often calculated using confirmatory factor analysis or exploratory factor analysis. Because this study contains only the 5 items measuring patient involvement, the ability to perform a factor analysis is limited. The omitted structural analysis means that this study cannot conclude whether the construct is 1D or how well it differentiates from other related aspects. This limitation highlights the need for future studies with a more comprehensive item pool to allow robust structural analyses and a more thorough examination of the construct’s dimensionality.

An analysis of responsiveness measures whether the constructed measure responds to a change in the underlying problem [25]. In this study, we do not have sufficient data to perform this analysis, so we cannot conclude how responsive the construction measure is.

In this study, PP partners in research were part of the analytical process, and 2 PP partners were included. This meant the data analysis incorporated a lay perspective and strengthened the analysis. The PP partners also articulated that a focus on user involvement in clinical practice is valuable, and they expressed how the clinic could use the PRO questions in clinical practice. Only 2 PP partners still participated in the analytical process. Increasing the number of PP partners and their involvement in other parts of the research process might have led to, for example, other lay perspectives or optimized the recruitment of participants. In future studies, we recommend more comprehensive involvement of PP partners [37].

In our study, the interview guide for the semistructured interviews focused on the entire short questionnaire. Hence, the focus was not entirely on the PRO questions, as the interviews covered all the questions in the short questionnaire. The quality of data concerning the semistructured interviews and the understanding of the 5 PRO questions could have been more in-depth if the 20 interviews had focused solely on the 5 PRO questions. Considering this, we conducted a second round of interviews with 5 participants. These interviews strengthened the validity of the study as they confirmed the findings from the first interviews [33,47].

The English translation of the Danish PRO questions has been guided by the protocol and guidelines from the International Society for Pharmacoeconomics and Outcomes Research (ISPOR) [48]. Although this makes the PRO measures usable in English-speaking countries, additional testing among adolescents in English-speaking countries would strengthen their validity regarding age, language, and culture. Here, cross-cultural validity, as described in the COSMIN checklist, is important to consider [24]. One of the items to address is whether the right people were involved in the translation process. Hence, it might strengthen the translation to include adolescents as part of the process. Furthermore, another aspect to consider is the cultural context of the health care system. Denmark is a high-income country, and its health care services are funded by an extensive welfare system, making them heavily or entirely subsidized. Furthermore, user involvement in care has been a focus for many years as an important factor in improving clinical outcomes [49]. This might affect clinical encounters and collaboration between adolescents and health care professionals and should also be considered when assessing generalizability beyond the Danish context. In clinical practice and research, we need tools to evaluate children’s and adolescents’ perspectives on user involvement in their care. One way to measure patient involvement is through PRO questions. This study has shown that the 5 PRO questions are valid for measuring adolescent involvement in their care, as the participants indicated that the PRO question items were easy to understand, relevant, and comprehensive. Still, when using the PRO questions, it is important to consider adolescents’ age, as the study shows that the PRO questions are most robust for those aged 15 years or older.

## Supplementary material

10.2196/86580 Multimedia Appendix 1 First and second interview guide.

10.2196/86580 Multimedia Appendix 2 Patient and public partners’ involvement in the analytical stage of the research.

10.2196/86580 Checklist 1 COSMIN checklist.

## Abbreviations

COSMIN: Consensus-Based Standards for the Selection of Health Measurement Instruments
HbA_1c_: hemoglobin A_1c_
ISPOR: International Society for Pharmacoeconomics and Outcomes Research
PP: patient and public
PRO: patient-reported outcome
WHO-5: World Health Organization–5
