# Supplementary material for: Investigation of the Content Validity, Feasibility, Internal Consistency, and Construct Validity of 5 Patient-Reported Outcome Questions on Patient Involvement in Care Among Adolescents With Type 1 Diabetes: Multimethods Study
Source: J Particip Med. 2026 May 19;18:e86580. doi: 10.2196/86580 (PMC13187346; doi:10.2196/86580)
Supplement: Multimedia Appendix 1 [file jopm-v18-e86580-s001.docx]

**Supplementary 1**

| Table 1 First and Second Interview Guide | |
| --- | --- |
| The Study: Investigating content validity, feasibility, internal consistency, and construct validity of five Patient-Reported Outcome (PRO) measures for patient involvement among adolescents with type 1 diabetes | |
| First interview guide | |
| Introduction:   - *Introduction:* The interview deals with 1) your experiences using the questionnaires in the consultation and the design of the questionnaires and 2) the supplementary treatment offer you have been offered (if any). - *Anonymity:* Your name will be anonymized. - *Audio recording:* The interview will be audio recorded and transcribed. - *Time:* The interview takes about 30 minutes. | |
| *Themes* | *Interview Questions* |
| Common part  *Introduction focusing on the use of questionnaires*  *The technical part - how was it, and how did you do it?*  *The content of the questionnaire*  *Experience in assessing and deciding on one's condition and needs*  *Use of the questionnaires in the consultation*  *(and the patient-involving potential)*  *TOBS about treatment efforts for those who have > 20 DEPS-R*  Common part  *End* | I would like to start by hearing if you have previously tried to answer questionnaires about how you feel (e.g., at school).  Do you remember how you were introduced to answering a questionnaire before your annual diabetes check?  Were you told why you had to answer the questionnaire before your diabetes check-up, or did you maybe read about it somewhere?  Why do you think that you should answer the questionnaire before the annual interview?  Try to tell me about the last time you had to answer a questionnaire before an annual interview.  What did you do? Where were you sitting? Which 'devise' do you answer (a phone, tablet)?  How long do you think it took? Do you think it took too long?  Were you alone when you answered? Did it require preparation?  Have you had technical problems answering the questionnaire and booking your annual interview (When you need to log in)?  You have answered this questionnaire about 1) how you feel (well-being), 2) your food, body, and weight, 3) involvement in your treatment, and 4) your overall assessment of your last visit to the hospital/diabetes check-up.  Are there any questions that are difficult/easy to understand or answer? For example, do you know what "energetic" means? Do you understand what the "normal range" is? Do you know what "ketones in the urine" are? Do you know what it means to "be consulted"? And what about "health professionals," "experiences," and "concerns"?  Are there any of the questions that you (or your parents) cannot understand we are asking?  Do you think some questions are missing? For example, if there is anything you are worried about? Or parts of your everyday life that take up space in relation to your diabetes, e.g., school or hobbies?  Do you think these are superfluous questions?  Have you used the comment field in the questionnaire for anything?  What has it been like to tell via a questionnaire how you feel about your diabetes?  How has it been like to assess your feelings concerning eating and diabetes treatment?  What has it been like to answer how you would like to be involved in your treatment?  The last time you were at the annual check-up, Could you tell us how the answer was used in the interview/consultation? (For example, did you talk about your eating or how you felt?  Do you remember any of the things you talked about? Did you talk about something new or different than usual? (For example, did you talk about decisions in relation to your treatment?)  (Only over 15 years :) Have you used Sundhed.dk to view your questionnaire responses before an interview? (If under 15 years of age: have you and your parents looked at the answers together before the consultation?)  Do you feel that you have learned something about your disease/diabetes by filling out the questionnaire before the annual check-up? (For example, have you gained a better understanding of your treatment or diabetes?) (= health-related reflection)  Is an annual check-up different when you have answered a questionnaire beforehand compared to if you have not?  You have been on a new course. Would you like to tell us what course (type of treatment) you have been on?  Which people have you met in the new course? (doctor, dietician, psychologist, psychiatrist, nurse, body therapist?)  Would you like to tell us when you were offered the new course?  Did you find at the time that you needed the offer  Who decided that you should have the offer?  Do you remember anything about the process (good/bad)?  Was there anything you missed in the process?  Did you have any influence on what you could participate in in terms of offers?  Did you actively work on yourself between visits (at the hospital/outpatient clinic)?  How did you experience the conversations with the different people in your course?  Did you participate in group offers?  What do you think about being in the group (was it good/bad)?  Would you prefer a different doctor or nurse who participated in the work on disordered eating than the one you knew from the outpatient clinic or hospital?  Did you talk about the questions afterward? If so, what questions?  Did you talk about other things before the annual check-up than you usually do? If so, which ones?  Do you have anything else you want to ask about before we finish?  Thanks for your help! |

| **Second interview guide** | |
| --- | --- |
| **Introduction and preparation:**  The interviews aim to examine the use of the five indicator measures for user involvement, the children's/young people's understanding of the questions, and the design of the questionnaire.  The five PRO questions are sent to the interviewees before the interviews and will be used as dialogue support.  The interviews are recorded (by agreement with the participant) and transcribed verbatim.  Participants are informed about anonymity and consent. | |
| **Themes** | *Interview Questions* |
| **Content of the questionnaire**  **Experience in assessing and deciding on own involvement**    **Common part**  ***End*** | Review the five indicator goals with the interviewee. Each question is read aloud, and the participant is asked to explain how they understand it.   1. The healthcare staff asked about my own experiences with my illness 2. I was able to talk to the healthcare professional about any questions or concerns I had 3. The healthcare professionals encouraged me to ask questions or talk about concerns 4. I was consulted when decisions were made about what was going to happen 5. I have had appropriate conversations with the healthcare professionals about how I best handle my illness   *- Do you know who the 'healthcare staff' is? Would it be better to call them 'the doctor and/or nurse'?*  *- Can you give an example of what it means to have 'experiences'?*  *- Can you give an example of a 'concern' or a 'question'?*  *- Can you give an example of what it means to 'be consulted'? What else could you call it?*  *- Are there any of the five questions you cannot understand we are asking?*  *- Do you think there is a lack of questions? Is there anything the questions do not come up with when we talk about your involvement/the conversation with the doctor and/or nurse?*  Response categories   - Don't know, not at all, to a lesser extent, to some extent, to a great extent, to a very high degree, not relevant to me  - Is there an appropriate amount of response categories? Why/Why not? *- For example, can it be difficult to distinguish between 'to a lesser extent' and 'to some extent'?*  Number of questions - Is there an appropriate number of questions? Why/Why not? *Are there any of the questions you experience that are like each other/superfluous (and possibly can be merged without much importance to you)?*  *For example, 'I talked to the healthcare staff about the questions or concerns I had' and 'The healthcare staff encouraged me to ask questions or talk about concerns' and 'I have had conversations with the healthcare staff to an appropriate extent about how best to manage my illness.'*  Response categories   - Don't know, not at all, to a lesser extent, to some extent, to a great extent, to a very high degree, not relevant to me  - Is there an appropriate amount of response categories? Why/Why not? *- For example, can it be difficult to distinguish between 'to a lesser extent' and 'to some extent'?*  Number of questions - Is there an appropriate number of questions? Why/Why not? *Are there any of the questions you experience that are like each other/superfluous (and possibly can be merged without much importance to you)?*  *For example, 'I talked to the healthcare staff about the questions or concerns I had' and 'The healthcare staff encouraged me to ask questions or talk about concerns' and 'I have had conversations with the healthcare staff to an appropriate extent about how best to manage my illness.'*  *- What has it been like to answer the five questions about the conversation with the doctor/nurse?*  *- Do you have anything to add?* Thank you for helping us. |
