# Supplementary material for: Investigation of the Content Validity, Feasibility, Internal Consistency, and Construct Validity of 5 Patient-Reported Outcome Questions on Patient Involvement in Care Among Adolescents With Type 1 Diabetes: Multimethods Study
Source: J Particip Med. 2026 May 19;18:e86580. doi: 10.2196/86580 (PMC13187346; doi:10.2196/86580)
Supplement: Multimedia Appendix 2 [file jopm-v18-e86580-s002.docx]

| Supplemental Table 2 Patient and public partners’ involvement in the analytical stage of the research | | | | |
| --- | --- | --- | --- | --- |
|  | Information given | Task | Communications path | Participants |
| Preparing for and starting the analysis | Seven word-files were mailed to the patient partners. One to six included codes based on the interviewed participants’ description of their understanding and interpretation of the six indicator measures. The seventh word-file encompassed codes outlining the interviewed participants’ experience with answering the short survey, how they valued the PRO questions, and the challenges with using them. A table of the PRO questions like Table 1  - Information on how to analyze the data. - Agenda for the meeting. - Information about the other participants attending the analytical meeting. | Reading the text in the seven word-files.Starting to analyze data based on the following questions:How do the adolescents in the interviews understand the six questions?Do they understand the questions equally?Are there words that they have difficulty understanding or do not understand?What challenges do you think the adolescents describe in relation to answering the short survey and the questions about involvement? | E-mail Communications | The two patient partners and the first authorThe two patient partners, the first, second, and last author. |
| The analytic meeting | The content of the meeting:   1. Welcome and presentation of participants. 2. Outlining the plan for the meeting. 3. Data analysis: One PRO question at a time, ending with the data describing the interviewed participants’ experience answering the short survey and the PRO questions.    1. First, the patient partner outlined her interpretation.    2. Then, the first author described how the other patient partner had described her interpretation in the e-mail.    3. Then, the rest of the participant asked questions and outlined their interpretation.    4. Then, a conclusion was made based on all participant contributions.   5. Evaluation of the process | All participants in the meeting participated in the analysis process by interpreting the data. Participants also had specific roles during the meeting.The first author leads the meeting, including asking supplementary questions and consolidating the conclusion.  - The patient partner provided her analysis of the code material. - The second author made observations and took notes during the meeting. - The last author focused on interpreting the patient partner’s analysis and providing the rest of the research team's analysis. | Video meeting with one patient partner and e-mail with the other patient partner |  |
