# Supplementary figures and images for: Investigation of the Content Validity, Feasibility, Internal Consistency, and Construct Validity of 5 Patient-Reported Outcome Questions on Patient Involvement in Care Among Adolescents With Type 1 Diabetes: Multimethods Study
Source: J Particip Med. 2026 May 19;18:e86580. doi: 10.2196/86580 (PMC13187346; doi:10.2196/86580)

**The COSMIN checklist**


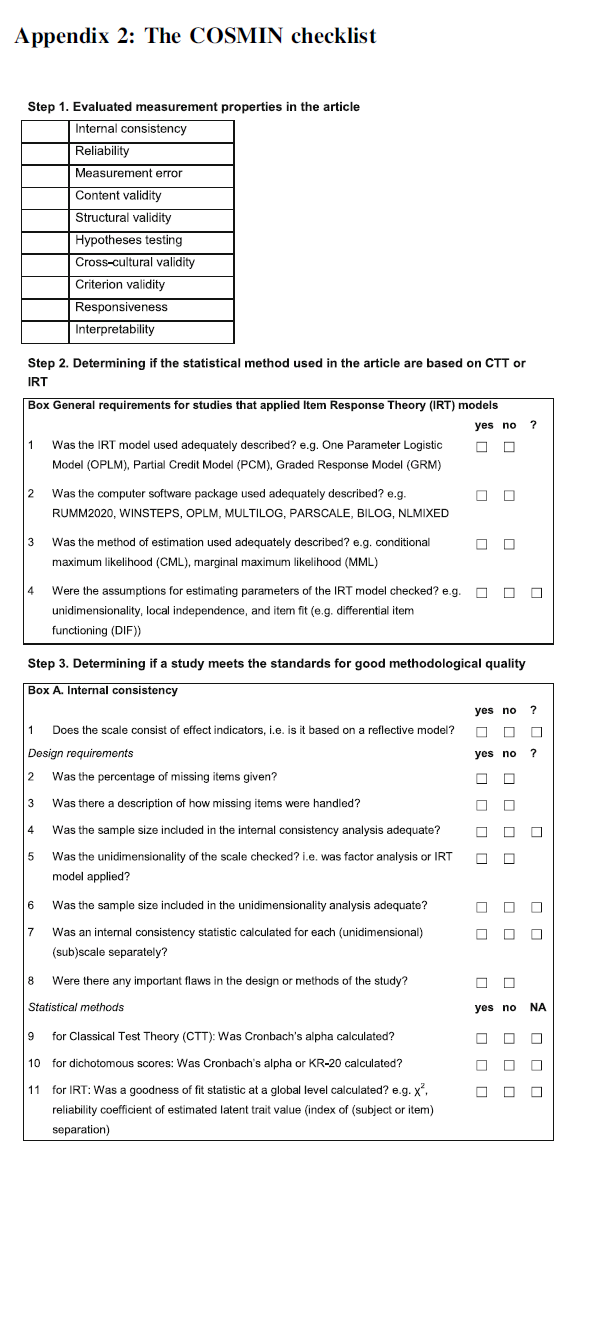


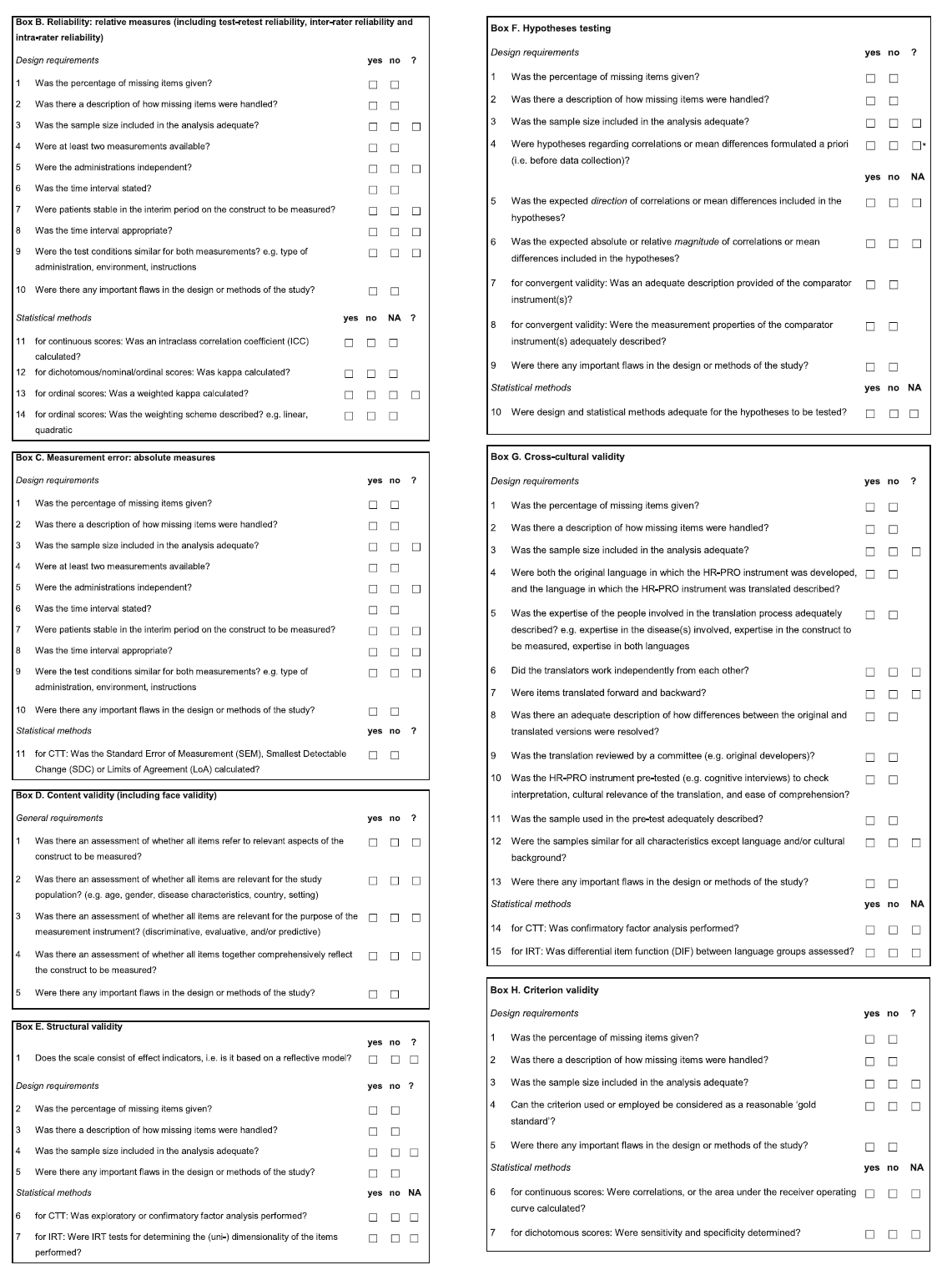


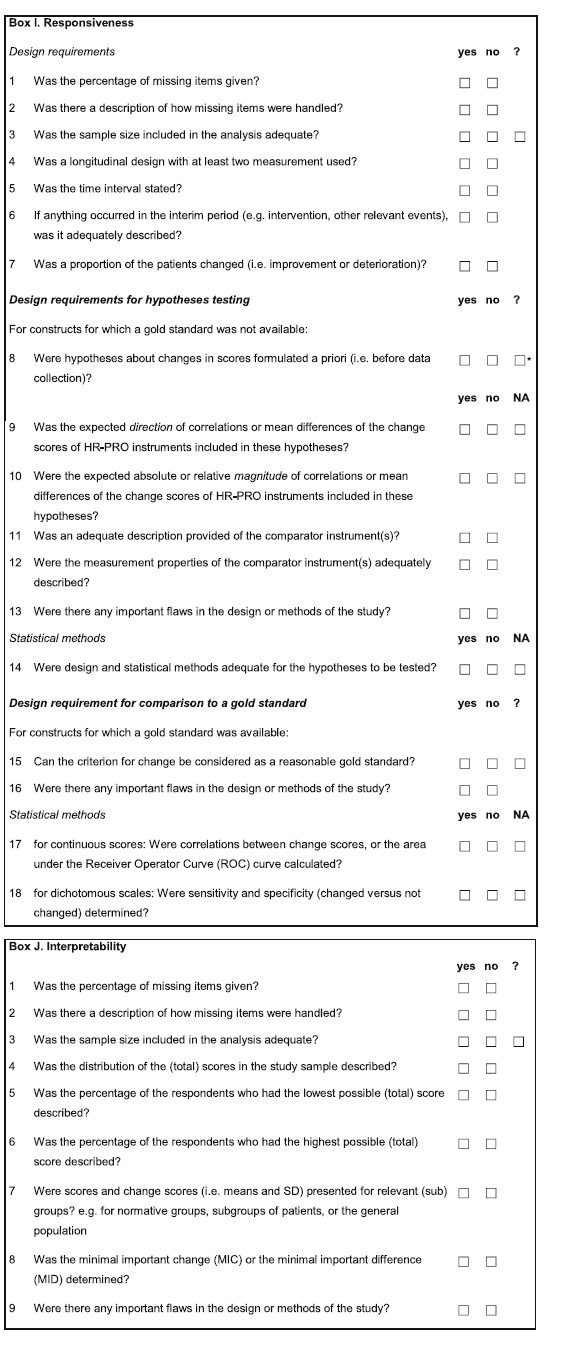

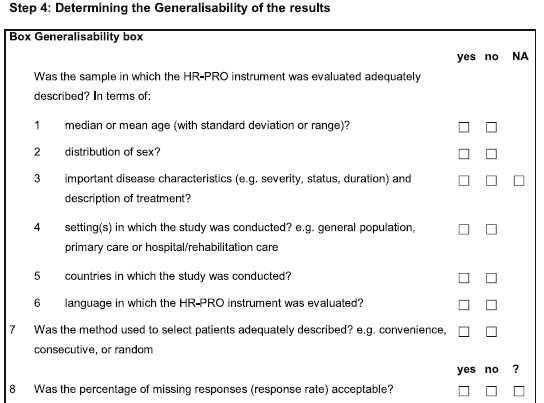

Supplement: Checklist 1 [file jopm-v18-e86580-s003.docx]
